# Does Stochastic and Modulated Wind Turbine Infrasound Affect Human Mental Performance Compared to Steady Signals without Modulation? Results of a Pilot Study

**DOI:** 10.3390/ijerph20032223

**Published:** 2023-01-26

**Authors:** Paweł Małecki, Małgorzata Pawlaczyk-Łuszczyńska, Tadeusz Wszołek, Anna Preis, Maciej Kłaczyński, Adam Dudarewicz, Paweł Pawlik, Bartłomiej Stępień, Dominik Mleczko

**Affiliations:** 1 Department of Mechanics and Vibroacoustics, Faculty of Mechanical Engineering and Robotics, AGH University of Science and Technology, 30-059 Krakow, Poland; 2 Department of Vibroacoustic Hazards, Nofer Institute of Occupational Medicine, 91-348 Lodz, Poland; 3 Institute of Acoustics, Faculty of Physics, Adam Mickiewicz University, 61-712 Poznan, Poland

**Keywords:** infrasound, low-frequency noise, wind turbine, effects on humans, infrasound playback

## Abstract

Wind turbines (WT) are a specific type of noise source, with unique characteristics, such as amplitude modulation (AM) and tonality, infrasonic and low frequency (LF) components. The present study investigates the influence of wind turbine infrasound and low frequency noise (LFN) on human well-being. In the between-subjects study design, 129 students performed a cognitive test evaluating attention and filled out questionnaires in three various exposure conditions, including background noise, synthesized LFN (reference noise) and registered WT infrasound (stimulus). No significant differences in test results or in the number of reported post-exposure feelings and ailments in various exposure conditions were found when analyzing them in males and females, separately. However, a significant association between pre-exposure well-being and reported post-exposure complaints was noted and explained by in-depth statistical analysis.

## 1. Introduction

Infrasound (IS) and low frequency noise (LFN) are ubiquitous in modern industry, the environment and urban lifestyle. The most common sources of infrasound are: traffic, large ventilation systems, public transport, wind farms, heat pumps and large machines [1,2]. Most of the reviews concerning the impact of infrasound on health have been based on data related to industrial workers or observations of areas exposed to infrasound due to their proximity to sources [3,4,5]. Such research is usually burdened with high ambiguity. For example, low-frequency audible components usually occurred during the exposure, which precluded an unambiguous answer to the question of whether the adverse effects can only be attributed to infrasound or audible bands. Accordingly, the most recent reviews of studies on the influence of infrasound on human health adopt conservatism while making conclusions about the adverse health effects directly caused by infrasound. Psychological and social mechanisms have been suggested as contributory factors to annoyance, which explains the observed adverse health effects better than exposure to very-low-frequency noise [6]. According to another report, about 10% of people living near infrasonic sources report general annoyance [7].

Most previously cited reports usually highlight the potential side effects, such as nausea, malaise, fatigue, undefined pain, sleep disturbance or irritability. However, there are also reports [8,9] signaling the potential use of infrasound in oncological therapy as support for the treatment with positive effects. A special case of infrasound is the phenomenon of binaural beats, which can be used in relaxation and sleep therapies [10,11], and the cited studies additionally indicate changes in the EEG (Electroencephalography) signal identical to exposure to infrasound.

Despite the indications regarding the effects of infrasound on mental health and cognitive functions in humans previously mentioned or reported in the literature, there are virtually no studies that directly investigate infrasound effects on human health in a randomized and controlled manner. In addition, so far there have been no studies analyzing the effect of infrasound on brain structure besides one piece of work [12] in which the effect of long-term human exposure to infrasound compared to a placebo was analyzed in a randomized manner. The presented study proves that long-term exposure (1 month) to infrasound with an amplitude above the values observed in wind farms and with a frequency of 6 Hz does not affect human behavior. This includes a number of variables related to health and psyche (i.e., self-assessment of noise sensitivity, sleep quality, psychosomatic symptoms or tension) and cognitive functions (i.e., alertness, constant attention, cognitive flexibility, divisive attention, attention shift and inhibition). At the same time, it has been observed that exposure to infrasound is associated with a decrease in gray matter in areas of the brain that are associated with somatomotor and cognitive functions, such as working memory (bilateral VIIIa cerebellum) and higher auditory processing (angular gyrus, BA39), including functions, such as speech intelligibility/production or semantic/lexical processing and reading. In another study on the influence of infrasound directly on the brain [13], it was noted that exposure to infrasound caused a change in the BOLD (blood oxygen level-dependent) signal in the primary auditory cortex and superior temporal gyrus. These are areas in the brain that are largely responsible for higher order auditory processing, such as language comprehension.

Wind turbines are a specific type of noise source, with an impact on large areas. The noise emitted by wind turbines does not resemble common industrial noise [14,15]. It has specific acoustic characteristics, such as amplitude modulation (AM) and tonality [16], as well as LFN and IS components, which can contribute to higher perceived annoyance [17,18]. Recently, Turunen et al. [19] carried out the first large-scale questionnaire study examining symptoms intuitively related to infrasound by people living near wind turbines in Finland. Nearly half of them reported ear symptoms; 26% cardiac symptoms; 24% headaches; 21% dizziness; 9% fatigue, high blood pressure or joint aches; and 7% nausea and difficulty focusing. In addition, 40% of symptomatic respondents reported negative effects on their health and 29% on their ability to work. The aforementioned study revealed that 70 out of 1351 respondents (5%) reported symptoms, which they attributed to infrasound from a wind farm. The symptomatic respondents lived closer to the wind farm than the asymptomatic respondents. Furthermore, they more often suffered from chronic diseases, complained about the annoyance of wind turbines and believed that wind turbines posed a health risk. Moreover, out of all the respondents, 10% considered wind turbine infrasound as a high risk to personal health and 18% as a high risk to health in general [19].

Although a great deal of research has been carried out over the years to evaluate adverse effects of different kinds of noise, it mostly concerns noise at rather high levels and/or occupational exposure, whereas studies of infrasound and LFN, in particular, at low SPL, are rather scarce [20,21]. Furthermore, most of the previous laboratory studies on the IS and LFN effects on cognition functions gave inconsistent results and did not allow the determination of noise threshold values above the level at which this effect occurs. For example, Moller [22] analyzed equal annoyance curves for pure tones in the frequency range of 4 Hz–31.5 Hz and found that when IS and LFN become audible, a slight increase in SPL leads to a large increase in annoyance. In turn, Persson et al. [23] compared annoyance related to LFN and noise without prominent content of low frequencies but at a similar A-weighted SPL and found that LFN was more annoying and more difficult to adapt. Similarly, Kjellberg et al. [24] investigated two types of noise with SPLs in the range of 49–86 dBA and frequencies from 15 and 50 Hz in twenty subjects. At the same A-weighted levels, LFN was perceived as 4–7 dB louder and 5–8 dB more annoying than higher frequency noise. Moreover, some previous studies generally indicated that LFN at levels that could occur in the occupational environment, including those typical for office-like areas and industrial control rooms (40–60 dBA), might be assessed as annoying and reduce the human mental performance, particularly when executing more demanding tasks [25,26,27]. Moreover, subjects classified as sensitive to noise might be at higher risk. 

Substantial attention has also recently been focused on investigating human responses to wind turbine noise. Laboratory experiments complement field surveys as they provide a more controlled environment needed to analyze causal relationships between characteristics of wind turbine noise and some of its effects [28]. According to a recent literature review by Karasmanaki [29], the effects of wind turbine noise on individuals’ health, sleep, cognitive performance and annoyance have been investigated by a significant number of experiments and listening tests. Even though these studies examine the impact of short-term rather than long-term exposure to wind turbine noise, they provide objective observations, which could be used to verify residents’ reports of WTN impacts recorded in quantitative research. However, only a few studies have, to date, been performed concerning the impact of wind turbine IS or LFN, while the majority of them focus on wind turbine noise in general. For example, such experiments were recently performed as part of a larger research project commissioned by the Finnish Government‘s Analysis Assessment and Research Activities [30]. They were aimed at the assessment of contributions of infrasound to the perception, annoyance and physiological reactions elicited by wind turbine sound. Sound samples recorded inside and outside residential houses near wind turbines with the highest infrasound levels and depth of AM were chosen for laboratory investigations. In the aforementioned experiments, the detectability and annoyance of both inaudible and audible characteristics of wind turbine noise were determined, as well as autonomic nervous system responses: heart rate, heart rate variability and skin conductance response. The participants were divided into two groups based on whether they reported experiencing wind turbine infrasound-related symptoms or not. It has been shown that people who have reported symptoms related to infrasound showed no increased sensitivity to wind turbine infrasound (i.e., they did not detect infrasonic contents of wind turbine noise). Total wind turbine SPL and amplitude modulation resulted in increased annoyance not infrasound. In turn, the wind turbine infrasound or wind turbine sound annoyance were not related to either heart rate or heart rate variability or to skin conductivity (physiological measures of stress). The presence of infrasound had no influence on the reported annoyance or the measured autonomic nervous system responses. No differences were observed between the two groups. These findings suggest that the levels of infrasound in the current study did not affect perception and annoyance or autonomic nervous system responses, even though the experimental conditions corresponded acoustically to real wind power plant areas.

The main aim of the current study is to investigate whether the IS and LFN accompanying the operation of wind turbines in Poland affect human well-being. In particular, an attempt has been made to answer the question of whether modulated IS and LFN can negatively affect mental performance compared to signals without modulation.

## 2. Methodology

### 2.1. Stimuli

The main goal of the experiment was to examine the IS and LFN generated by wind turbines, with the first step being to accurately capture the proper stimuli for the experiment. First, preliminary recordings and sound pressure measurements were conducted in the Kościuszko ventilation shaft of the Wieliczka salt mine near Krakow, Poland in order to verify the usefulness of the planned recording equipment in the measurements of IS and LFN. This source was chosen because it generates low-frequency band noise regardless of the wind conditions.

**Figure 1:**
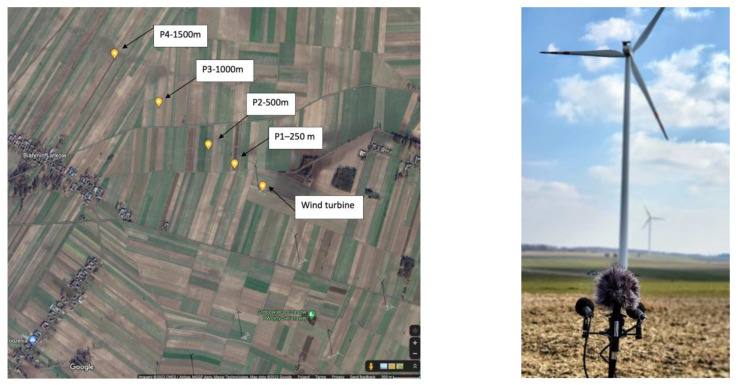
The wind turbine noise measurement and recording points. Satellite photo source: google.maps.com (accessed on 23 June 2022).

The in situ recordings were conducted on wind farm E (anonymous due to the agreement with the farm operator) at a distance of 130 m from the turbine on 9 July 2021 and on farm A at a distance of 250 m from the turbine shown in Figure 1. Due to the more stable weather conditions, since there was no wind on the microphones’ membranes during all turbine nominal work for at least 10 min, recordings from wind farm A were used in the following parts of the experiment.

The previously recorded wind turbine noise was filtered with a finite impulse response low pass filter in order to obtain IS only. The passband frequency was set to 20 Hz, while the stopband was 22 Hz with 90 dB attenuation using the Kaiser window design method.

### 2.2. Apparatus

The experiment (stimuli exposition) took place in the public address (PA) audio equipment warehouse in Krakow during audio engineering classes. The experiment location was chosen due to several factors. It was equipped with a set of industry standard JBL VTX G28 subwoofers (1.5 × 1.5 × 0.5 m each) that allowed high levels of low frequencies to be generated. In addition, the warehouse was quite big (12 × 30 m) and high (from 4 to 7 m) and was made of light walls consisting of steel beams, metal sheets and thin insulation. This was an important factor due to the potential standing waves that can be profound and uncontrolled in a hard-wall scenario. The warehouse background G- and A-weighted (according to ISO 7196:1995 and IEC 61672-1:2013 shown in Figure S1) equivalently continuous sound pressure levels (SPL) were approx. equal to 62 dBG (L_Geq_) and 35 dBA (L_pAeq_), respectively. During the experiment, two subwoofers were used and the participants were situated in front of the covered subwoofers at approx. 3–7 m in an area of around 15 m^2^.

The following equipment was used for the sound recording:

- A DPA 4006 pre-polarized condenser, pressure microphones with windscreens in AB stereo configuration with the effective frequency range ±2 dB: 10 Hz–20 kHz, sensitivity of 40 mV/Pa and equivalent thermal noise level of 15 dBA re. 20 µPa.

A ZOOM F8n field recorder, with 8 microphone inputs of equivalent input noise of −127 dBu or less (A-weighted, +75 dB input gain, 150 Ω input), and a frequency response given by the manufacturer of 20 Hz to 60 kHz, +0.5 dB/−1 dB (192 kHz sample rate). The ZOOM F8n measurement of frequency response performed by the authors showed that the lower limit is evenly expanded to 10 Hz and only falls by −3 dB to 5 Hz. The actual lower limit without attenuation of the measured microphones is at around 3–4 Hz.

- The level calibration of stimuli playback was performed in situ using a SVAN 959 sound analyzer equipped with a G.R.A.S. 40AE microphone and SV12L microphone preamplifier. The device is of class 1 accuracy, in accordance with EN 61672-3:2014, so the monitored level error should not exceed +/− 1.1 dB.

The target level of stimuli to be played back by the subwoofer set was established based on measurements in wind farm A. The electric power of the WT is 2 MW, the recordings and the measurements were conducted at a height of 1.6 m and 250 m distance leeward. The measured SPL equaled 80.3 dBG and 46.3 dBA. The modulation depth of the recorded signal was around 4 dB and 1 Hz rate, and the signal level, as well as particularly the frequency bands, was very variable with deviations of approx. 10 dB.

**Figure 2:**
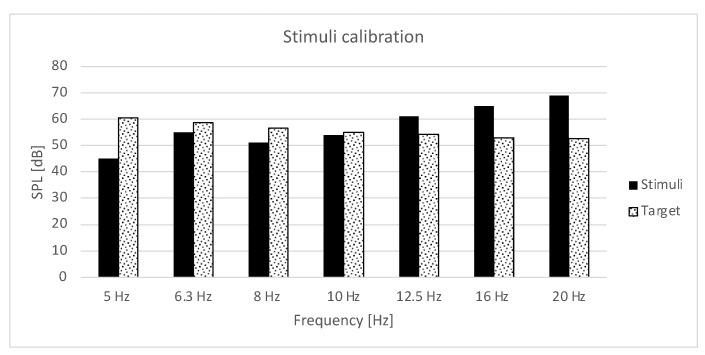
The target level of the wind turbine noise and the resulting stimuli after calibration.

The stimulus signal was amplified and equalized to achieve levels in 5–20 Hz bands as close as possible to target levels in the corresponding frequency range. The result of the level calibration is shown in Figure 2. The proper match of levels was achieved for the 10 Hz band only. For 8 Hz, the level of stimuli was around 6 dB lower, for 6.3 Hz it was around 3 dB, and for 5 Hz it was significantly lower than the target level. For these very low frequencies, the subwoofers could not produce enough energy and any more equalization caused audible harmonic distortion and an even more prominent rise of levels in 10–20 Hz frequency bands. Any further cuts in the bands over 10 Hz caused dumping of the lower frequencies. The overall L_Geq_ level of the stimuli was around 3 dB higher than that observed in the field due to the calibration issues. The resulting spectrum of stimuli is a kind of compromise between the target level of IS around wind turbines and technical limitations of the sound source.

The levels of stimuli were measured in the area where the stimuli exposure took place and were monitored all the time during the experiment.

In general, three different noise exposure conditions were used in this experiment:

- “Stimulus”, i.e., recorded and filtered wind turbine noise at an approx. equivalent-continuous G-weighted SPL (L_Geq_) and low-frequency (LF) A-weighted SPL (L_pA,LF_) equal to 83 dBG and 47 dBA, respectively;
- “No stimulus”, i.e., background noise at approx. 63 dBG/43 dBA;
- “Reference signal”, i.e., synthesized steady LFN at approx. 78 dBG/46 dBA.

**Figure 3:**
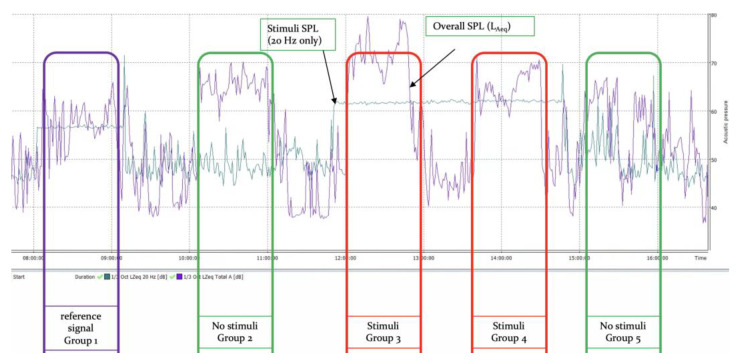
Level of stimuli and acoustic background monitoring during a sample day of the experiment.

The exemplary plot of SPLs during the daily sessions is shown in Figure 3. The figure shows time slots when exposition took place with randomly applied stimuli (wind turbine IS noise), the reference signal or none. During the classes, the overall SPL (L_Aeq_) was high but not related with stimuli or the reference signal level. It was the background noise during the experiment that was a result of outdoor urban sounds (traffic, etc.), talks between the students and teacher, and occasional audio signals generated during work with microphones and mixers. There was no intentional or artificial noise introduced in the case of background noise exposure only. As a reference signal, a set of pure tones in IS 1/3 octave bands was used. A total of 7 sine oscillators (5–20 Hz) were used to synthesize the reference signal without any AM or deviation. The reference signal level was adjusted to the same level as the stimuli. The background levels in the acoustic range were much higher during the classes, as expected, but some variations of background noise below 6 Hz were observed, so it is subject to additional statistical analysis of the results.

### 2.3. Participants

The study comprised 15 seminary groups of students (129 subjects, including 74 females and 55 males), aged 21–24 years. The experiment was performed during audio engineering classes that lasted approx. 70–80 min. The number of examined student groups is the result of the availability of the experiment venue for classes and the experiment. The group size varied from 8 to 12 participants. All of the participants reported normal hearing, which is consistent with their field of study, being acoustics.

Since a between-subjects study design was applied, each group of students was asked to perform during randomly assigned noise exposure conditions, since a between-subjects study design was applied, each group of students was asked to perform a cognitive test evaluating attention, after approx. 70–80 min of audio engineering classes during randomly assigned noise exposure conditions.

Participation in the study was voluntary and there was no financial gratification for the participation. Subjects were recruited using an oral advertisement. No exclusion criteria were applied; thus, all the people who responded to the invitation could participate. The subjects certified in writing their consent to participate in the research. The study design and methods were approved by the Ethics Committee for the Research Involving Human Participants at the Adam Mickiewicz University in Poznan (Ordinance No. 15/2020/2021 adopted on 28 September 2021) and the Bioethics Committee of the Nofer Institute of Occupational Medicine of Lodz, Poland (Decision No. 4/2022 of 10 June 2022).

A number of study subjects (*n* = 64) were exposed to recorded and filtered wind turbine noise (“stimulus”), with the others exposed to “no stimulus” (*n* = 43) or to the “reference signal” (*n* = 22).

### 2.4. Procedure

**Figure 4:**
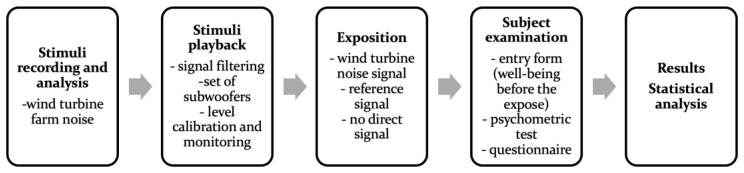
The experiment design.

The overall experiment concept is presented in Figure 4, which shows all the phases described in detail in the previous and following subsections.

In order to elucidate the influence of infrasound and LFN on cognitive functions, a Work under Stress Simulator (SPS) paper and pencil test was selected. This test was created as a result of the need for a test examining the impact of distractors (stressors) on the efficiency of cognitive functions, especially the system responsible for the selection of information. Attention plays such a role in our cognitive “system”. Normally, it is used with a device consisting of a table with a light-permeable top, on which we place the test sheet, headphones and a remote control to control the device. During the test task (number substitution test), the subject is exposed to stimuli that make it difficult to perform it correctly. It takes 3 min to complete one task. The SPS simulator allows researchers to determine the level of fitness and the degree of concentration on a task under stress caused by disturbing stimuli (light and sound). However, in this study, the three different exposure conditions play the role of the disturbing stimuli. The test result is the number of correct answers [31].

Prior to performing the psychometric test, the study subjects were asked to fill in a so-called “initial” (pre-exposure) questionnaire developed to enable the evaluation of their well-being before classes. The aforementioned questionnaire included the following questions:

(1) How many hours did you sleep last night?
(2) Did you have any problems falling asleep?
(3) If YES, why?
(4) Was the sleep broken?
(5) Was the sleep as long as usual?
(6) Did you wake up rested?
(7) How do you feel now? (Very good/ Good/So-so/Rather bad/Bad)
(8) Do you have health problem now?
(9) If YES, then what is it? …

In turn, after the psychometric test, they answered a “final” (post-exposure) questionnaire concerning symptoms (feelings and complaints) subjectively related to acoustic conditions:

(1) Did you feel any additional or unusual sensation during the classes?
(2) Did you hear additional sounds?
(3) Did you feel pressure in your ears?
(4) Did you feel pressure in your head?
(5) Did you feel vibration in the room?
(6) Did you feel vibration in part of your body?
(7) Did you suffer in any way?
  (7a) Headache?
  (7b) Problems with concentration?
  (7c) Dizziness?
  (7d) Drowsiness?
  (7e) Fatigue?
  (7f) Others …?

According to the post-exposure questions, any later feelings refer to questions 1–6, while ailments refer to questions 7–7f. The questionnaire was modeled on a previously developed one, which was aimed at evaluating the effects of the exposure to LFN [25].

### 2.5. Data Analysis—Main Hypothesis

In order to analyze the possible impact of IS/LFN on human well-being, the study subjects were divided into subgroups according to exposure conditions and gender, since it was noticed during data evaluation that there are some significant differences between males’ and females’ responses to the experiment. This also corresponds with the literature findings that the prevalence of noise annoyance was higher among women than in men [32,33,34]. In the study of Okonon et al. [32], the authors found that females showed some evidence of an association with noise annoyance and stronger evidence of association with noise sensitivity than males.

The majority of answers to the questionnaires shown in Section 2.4 were YES or NO, while only a few were given on the ordinal scale (e.g., on a 5-grade verbal rating scale). However, additionally, the total number of feelings and ailments subjectively related to exposure conditions was also determined in case of the post-exposure questionnaire. Thus, the above-mentioned answers (YES or NO) were presented as proportions with 95% confidence intervals (95% CI) in various subgroups of students. The differences between them were compared in pairs using the exact Fisher test or chi^2^ test.

One-way ANOVA, or its non-parametric equivalent, i.e., the Kruskal–Wallis H test, where applicable, was used to evaluate the main effect of exposure conditions on the psychometric test results and other variables (e.g., the total number of ailments) in females and males, separately and together. On the other hand, the differences between exposure groups were compared in pairs using a post hoc Tukey’s HSD test or multiple comparisons using the rank sums method in the case of non-parametric data.

On the other hand, the strength and direction of associations existing between variables were assessed using a gamma coefficient. To evaluate differences between two unmatched samples of observations on an ordinal scale (e.g., comparing the answers of men and women on a 5-grade verbal rating scale), the Mann–Whitney test was used.

The Statistica (ver. 9.1. StatSoft Inc., Tulsa, OK, USA) software package was used for statistical analysis. All tests were conducted with an assumed *p* = 0.05 significance level. However, when exploring several comparisons in pairs at the same time, to avoid the risk of mass significance, Bonferroni’s method was applied, reducing the *p*-value considered statistically significant by dividing it with the number of possible comparisons.

### 2.6. Additional Analysis

For an in-depth exploration of the main problem of the paper, additional analyses were performed in order to address the influence of known issues during the experiment mentioned in the method section, such as perceived changes in exposure conditions due to the mechanical movements of the loudspeaker coil, the sensed impact of exposure conditions due to the fatigue of subjects before the exposure or the influence of background noise below 5 Hz that was not possible to control. In order to exploit that, the binary logistic multiple regression was used to study the impact of the mentioned variables on the main hypothesis results. The Nagelkerke pseudo—R^2^ was applied as a measure of explained variance while the correct classification rate (CCR) was considered as a measure of fit of the logistic model. The results of the additional data analysis are presented in the Supplementary Materials.

## 3. Results

The subjective assessments of well-being before audio engineering classes in study subjects are presented in Table 1. There were no significant differences between subjects performing the psychometric test in various exposure conditions. Basically, with one exception, similar relations were observed when comparing answers given by females and males. It turned out that an almost two-times greater proportion of men than women woke up well rested before the classes (Table 1).

The outcomes of the post-exposure questionnaire are given in Table 2. There were no significant differences in answers to the questions between students exposed to the stimulus and reference signal. However, only some symptoms were more frequently reported by subjects exposed to the stimulus compared to those without any stimulus. Such relations were noted in the case of feeling the pressure changes in someone’s head and experiences of physical or mental discomfort, as well as the perception of any changes in exposure conditions (exact Fisher test, *p* < 0.05/3).

Furthermore, there were significant differences between females and males. Men more often than women reported pressure changes in their ears and felt vibrations in the room, while females generally more frequently sensed the impact of exposure conditions and complained of headache, sleepiness and fatigue (*p* < 0.5/3). Moreover, the total number of ailments related to exposure conditions was significantly greater in females than in males (Table 2). However, no significant impact of exposure conditions was observed when analyzing the proportions of answers to the post-exposure questionnaire in men and women separately (*p* > 0.05/3).

The analysis, using the gamma coefficient, revealed a significant relationship between pre-exposure well-being and reported post-exposure complaints(Table 3). In particular, it has been shown that the greater the number of hours being slept or the better well-being before the classes, the smaller the number of reported post-exposure ailments. Furthermore, subjects with health problems suffered from a greater number of ailments subjectively related to exposure conditions, while those with problems falling asleep (or woke up rested) reported a greater number of feelings due to exposure conditions compared to others (Table 3).

**Figure 5:**
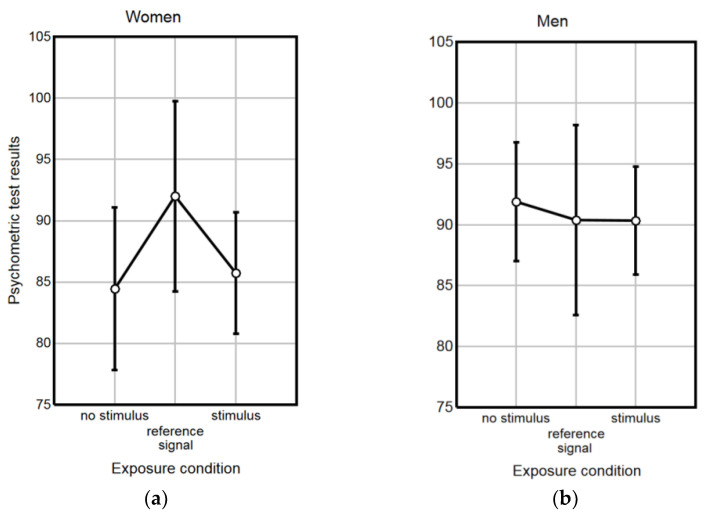
Results of the work under stress simulator test in various exposure conditions among (**a**) female and (**b**) male students. Data are given as mean values with 95% confidence intervals.

No significant associations between the performance level of the psychometric test and the self-assessment of students’ well-being before classes were noted. However, there was a weak but statistically significant positive relationship between gender and performance level of the psychometric test (γ coefficient = 0.212, *p* < 0.05). Therefore, the differences in the psychometric test performance due to various exposure conditions were analyzed both in females and males, separately and together. The results in Figure 5 show that no significant effect of exposure conditions was noted in females (ANOVA, F(2, 70) = 0.125 *p* = 0.883) or males (ANOVA, F(2, 50) = 1.246 *p* = 0.296). A similar outcome was obtained when analyzing the impact of exposure conditions in all study subjects (ANOVA, F(2, 123) = 0.403 *p* = 0.669).

**Figure 6:**
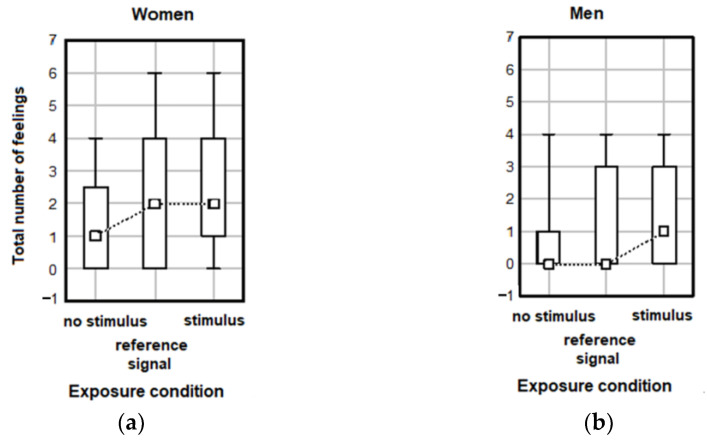
Total numbers of post-exposure feelings reported in various exposure conditions by (**a**) female and (**b**) male students. Data are given as median values with 5th, 25th, 75th and 95th percentiles.

**Figure 7:**
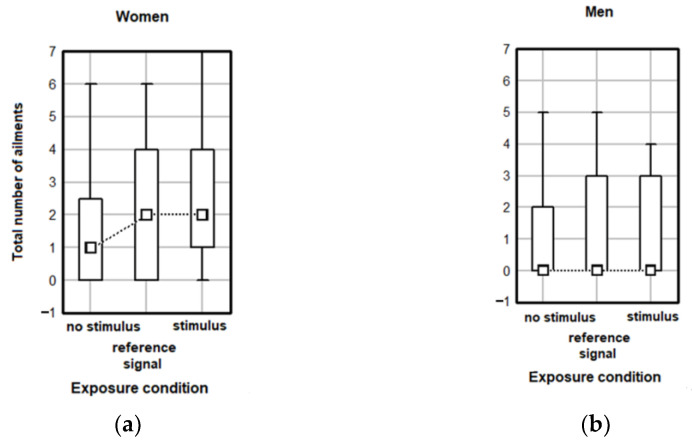
Total numbers of post-exposure ailments reported in various exposure conditions by (**a**) female and (**b**) male students. Data are given as median values with 5th, 25th, 75th and 95th percentiles.

The gender-related analysis was also performed for the total number of feelings and the total number of ailments in relation to exposure conditions. The results show no significant effect of exposure conditions for females (Kruskal–Wallis test H(2, N = 74) = 3.91 *p* = 0.142) or males (Kruskal–Wallis test H(2, N = 55) = 1.545 *p* = 0.462), considering the perception of stimuli (Figure 6) and no significant effect of exposure conditions was noted in females (Kruskal–Wallis test H(2, N = 74) = 2.442 *p* = 0.259) or males (Kruskal–Wallis test H(2, N = 55) = 0.931 *p* = 0.628) in regard to the total number of ailments (Figure 7). Similar conclusions was drawn when analyzing the impact of exposure conditions on the total number of feelings (Kruskal–Wallis test H(2, N = 129) = 5.251 *p* = 0.072) and ailments (Kruskal–Wallis test H(2, N = 129) = 2.276 *p* =0.320) in females and males together.

## 4. Discussion

In this paper, all stages of the experiment were described, starting from the recording of the IS and LFN generated by wind turbines and their playback using a set of PA grade subwoofers and ending with a discussion of the results. The recording of the infrasound is not a complex procedure, but it requires careful study of the recording equipment below the 20 Hz range as most of the conventional audio recording devices very often have a built-in HPF (high pass filter). On the other hand, IS playback is a very difficult process. In order to achieve proper levels in the IS range, very high loudspeaker membrane amplitudes are required. Most of the conventional audio equipment is not efficient below 20 Hz, and increasing these bands causes prominent harmonic distortion or mechanical noise accompanying IS playback. Hence, the exact recreation of wind turbine IS was not possible for the selected bands below 10 Hz. Attempts at the exact matching of the stimuli to the wind turbine signals resulted in rising levels of higher harmonics due to the distortion. Therefore, the frequencies at around 20 Hz were much higher during playback than expected and, as a result, caused more frequent stimuli discrimination than predicted. In particular, the statistical analysis of the results showed that this phenomenon did not disturb the credibility of the results. Another potential technical obstacle with IS and LFN exposition is the standing wave issue. The length of low frequencies very often corresponds with the building, room or corridor dimensions, and high local resonances or antiresonances are observed. This problem was solved by conducting the experiment in a lightweight building construction. Another potential solution could be working outside the building, but this would cause some other technical problems as well.

However, the conducted experiment has several weaknesses as well. Firstly, between-subjects study design was selected with an unequal number of participants. Secondly, the inference was based on the results of one psychological test and two questionnaires. Thirdly, individual sensitivity to noise was not taken into account and, fourthly, basically no exclusion criteria of participants were used. Meanwhile, in the case of the between-subjects study design: different people test each condition, so that each person is only exposed to a single user interface. On the other hand, in the within-subjects (or repeated-measures) study design: the same person tests all the conditions (i.e., all the user interfaces). The between-groups designs reduce learning effects; repeated-measures designs require fewer participants and minimize the random noise. On the other hand, increasing the number of people surveyed may make it possible to recognize smaller differences between the surveyed groups as statistically significant. In turn, the unification of group sizes may lead to homogeneous variances in groups, which would enable statistical analyses that are unavailable at the present stage of research, e.g., the analysis of covariance (ANCOVA).

Nevertheless, generally speaking, the outcomes of the present study do not contradict the results of previous research. However, further studies are needed before firm conclusions can be formulated concerning the health impacts of wind turbine infrasound, taking into account standardized and more appropriate psychological methods, such as more demanding cognitive tests.

## 5. Conclusions

In the current work, the influence of acoustic conditions and gender on the level of human mental performance, as well as that of the feelings and ailments associated with the exposure conditions, were analyzed.

The main, but not straightforward, conclusion of the work is that there were no statistically significant differences in response rates between subjects exposed to infrasound of WT origin and steady IS without AM modulation. However, small but significance differences were visible between people exposed to WT infrasound and people without exposure. Generally, the latter subgroup less frequently reported feeling pressure changes in the head, experience of physical or mental discomfort and the perception of any changes in exposure conditions. The second output should be especially robust due to its potential prominence; therefore, several other factors have been carefully examined.

There were also significant differences between females and males. Generally, a greater proportion of males perceived changes due to exposure conditions, while females more often felt worse after classes. However, no significant impact of exposure conditions was observed when analyzing the proportions of answers to the post-exposure questionnaire in men and women separately (*p* > 0.05/3).

There were no significant differences in the self-assessment of well-being before classes between subjects performing the psychometric test in various exposure conditions. Basically, with one exception, neither exposure conditions nor gender had a significant impact on the self-assessment of subjects’ well-being before classes. In addition, there were no significant associations between the performance level of the psychometric test and the self-assessment of students’ well-being before classes. On the other hand, a significant gamma coefficient between pre-exposure well-being and reported post-exposure complaints has been found. Generally, subjects well rested before classes felt better after their end. Additionally, no significant differences in performance levels of the work under stress simulator test in various exposure conditions were found in males and females analyzed separately. Similar results were obtained when analyzing the total number of feelings and ailments subjectively related to exposure conditions during classes.

Returning to the main conclusion and the expressed doubts, on the basis of the above additional factors and results analyses shown in the Supplementary Materials (Tables S1–S3), we conclude that it is much more probable that the obtained influence of WT IS on subjects’ well-being is a result of: unintentional perception of the stimuli, presence of IS background below 5 Hz or the tendency of a specific group (in this case females) to report negative well-being after the classes if they were tired before the classes.

## Figures and Tables

**Table 1:** Answers to the pre-exposure questionnaire in study subjects divided into subgroups according to exposure conditions and gender.

Answers to the Questionnaire	Exposure Conditions			Gender	
	No Stimulus (*n* = 43)	Reference Signal (*n* = 22)	Stimulus (*n* = 64)	Females (*n* = 74)	Males (*n* = 55)
	M ± SD				
Number of hours slept	6.4 ± 1.3	5.8 ± 1.3	6.5 ± 1.6	6.2 ± 1.5	6.6 ± 1.4
Self-assessment of well-being on 1–5 score scale	3.6 ± 0.9	3.5 ± 1.0	3.6 ± 0.9	3.5 ± 1.0	3.7 ± 0.7
	**Proportion of Respondents [%]** **(95% CI)**				
Having trouble falling asleep	18.6 (9.6–33.0)	13.6 (4.1–34.4)	29.7 (19.9–41.9)	25.7 (17.1–36.8)	20.0 (11.5–32.6)
Sleep was interrupted	37.2 (24.4–52.2)	27.3 (13.0–48.5)	25.0 (16.0–37.0)	29.7 (20.5–41.0)	29.1 (18.8–42.3)
Sleep lasted as usual	53.5 (38.9–67.5)	27.3 (13.0–48.5)	37.5 (26.7–49.8)	36.5 (26.5–47.9)	47.3 (34.7–60.2)
Woke up refreshed	44.2 (30.5–58.9)	27.3 (13.0–48.5)	42.2 (30.9–54.4)	29.7 * (20.5–41.0)	54.5 * (41.5–66.9)
Having health problems now	18.6 (9.6–33.0)	27.3 (13.0–48.5)	18.8 (11.0–30.2)	16.2 (9.4–26.5)	25.5 (15.8–38.5)

CI—confidence interval. * Significant difference between females and males (chi^2^ test, *p* < 0.05).

**Table 2:** Answers to the post-exposure questionnaire in study subjects divided into subgroups according to exposure conditions and gender.

Answers to the Questionnaire	Exposure Conditions			Gender	
	No Stimulus (*n* = 43)	Reference Signal (*n* = 22)	Stimulus (*n* = 64)	Females (*n* = 74)	Males (*n* =55)
	Proportion of Respondents [%] (95% CI)				
Felt an additional signal, stimulus or other unusual sensation during the classes	11.6 (4.7–25.1)	9.1 (1.5–29.3)	26.6 (17.3–38.6)	18.9 (11.6–29.5)	18.2 (10.1–30.6)
Heard noise, a hum or sound other than the typical background acoustics	39.5 (26.4–54.5)	31.8 (16.3–52.9)	42.2 (30.9–54.4)	44.6 (33.8–55.9)	32.7 (21.8–46.0)
Felt pressure changes in the ears	25.6 (14.9–40.4)	22.7 (9.9–44.0)	29.7 (19.9–41.9)	20.3 ** (12.6–31.0)	36.4 ** (24.9–49.6)
Felt pressure changes in the head	9.3 * (3.2–22.3)	9.1 (1.5–29.3)	29.7 * (19.9–41.9)	17.6 10.5–28.0)	21.8 (12.9–34.6)
Felt vibrations in the room	7.0 (1.8–19.5)	22.7 (9.9–44.0)	21.9 (13.4–33.6)	10.8 ** (5.4–20.2)	25.5 ** (15.8–38.5)
Felt vibrations in the body	9.3 (3.2–22.3)	22.7 (9.9–44.0)	21.9 (13.4–33.6)	17.6 (10.5–28.0)	18.2 (10.1–30.6)
Experienced physical or mental discomfort	14.0 * (6.3–27.8)	27.3 (13.0–48.5)	34.4 * (23.9–46.7)	25.7 (17.1–36.8)	27.3 (17.3–40.4)
Headache	18.6 (9.6–33.0)	4.5 (−0.7–23.8)	17.2 (9.8–28.5)	21.6 ** (13.7–32.4)	7.3 ** (2.5–17.9)
Concentration problem	23.3 (13.1–38.0)	27.3 (13.0–48.5)	39.1 (28.1–51.3)	37.8 (27.7–49.3)	23.6 (14.3–36.5)
Dizziness	2.3 (0.0–13.4)	9.1 (1.5–29.3)	6.3 (2.1–15.6)	2.7 (0.2–10.0)	9.1 (3.6–20.1)
Sleepiness	30.2 (18.6–45.2)	36.4 (19.8–57.2)	35.9 (25.3–48.2)	41.9 ** (31.3–53.3)	23.6 ** (14.3–36.5)
Tiredness	44.2 (30.5–58.9)	36.4 (19.8–57.2)	39.1 (28.1–51.3)	54.1 ** 42.8–64.9)	21.8 ** (12.9–34.6)
Perceived some changes in exposure conditions	23.3 * (13.1–38.0)	31.8 (16.3–52.9)	48.4 * (36.7–60.4)	35.1 (25.3–46.5)	40.0 (28.1–53.2)
Sensed the impact of some exposure conditions	41.9 (28.4–56.7)	40.9 (23.3–61.3)	48.4 (36.7–60.4)	52.7 ** (41.5–63.6)	34.5 ** (23.4–47.8)
	**M ± SD**				
The total number of feelings subjectively related to exposure conditions	0.8 ± 1.1	1.0 ± 1.6	1.4 ± 1.5	1.1 ± 1.3	1.2 ± 1.5
The total number of ailments subjectively related to exposure conditions	1.4 ± 1.6	1.7 ± 2.1	2.0 ± 1.9	2.1 ± 1.9 ***	1.3 ± 1.8 ***

CI—confidence interval; M—mean; SD—standard deviation. * Significant differences between groups of students non-exposed and exposed to stimulus (the exact Fisher test, *p* < 0.05/3); ** Significant differences between females and males (chi^2^ test, *p* < 0.05). *** Significant difference between females and males (U-Mann–Whitney test, *p* < 0.05).

**Table 3:** Relationships between results of pre- and post-exposure questionnaires assessed using a gamma coefficient. Data concern all study subjects.

Answers to the Questionnaire		Total Number of	
		Feelings	Ailments
		γ coefficient	
1	Number of hours slept	−0.019	−0.226 *
2	Problems with falling asleep	0.423 *	0.301 *
4	Interrupted sleep	0.114	0.200
5	Sleep lasted as long as usual	0.229 *	−0.161
6	Woke up rested	0.218 *	−0.399 *
7	Self-assessment of well-being	−0.173	−0.500 *
8	Having health problems	0.006	0.435 *
	Tired before classes	0.091	0.408 *

* Significant values of the γ coefficients (*p* < 0.05).

## Data Availability

The data presented in this study are available on request from the corresponding author. The data are not publicly available due to the privacy of the participants.

## Supplementary Materials

The following supporting information can be downloaded at: https://www.mdpi.com/article/10.3390/ijerph20032223/s1, Table S1: Relationships between gender and outcomes of the post-exposure questionnaire assessed using a gamma coefficient. Data concern all study subjects; Table S2: Association between PC (any perceived changes in exposure conditions) or SI (the sensed impact of exposure conditions on well-being) (dependent binary variable) and gender, fatigue and type of noise conditions (independent variables) tested using logistic regression; Table S3: A gamma coefficient between the noise parameters and the outcomes of the post-exposure questionnaire. Figure S1: Nominal G-, A-, C- and Z-weighting characteristics according to ISO 7196:1995 and IEC 61672-1:2013.
