# Supplementary material for: Does Stochastic and Modulated Wind Turbine Infrasound Affect Human Mental Performance Compared to Steady Signals without Modulation? Results of a Pilot Study
Source: Int J Environ Res Public Health. 2023 Jan 26;20(3):2223. doi: 10.3390/ijerph20032223 (PMC9916344; doi:10.3390/ijerph20032223)
Supplement: Supplementary file 1 [file ijerph-20-02223-s001.zip › ijerph-2098223-supplementary.pdf]

Supplementary Material

The Supplementary Material shows the additional analysis of the obtained data in order to learn about the influence of known issues during the experiment on the results. For this purpose, some logical data aggregations have been made and additional binary variables have been introduced:

- subjects who slept for less than 5 hours, rated their well-being below 4 on a 1-5 scale or complained of any ailments, such as nausea, headaches, etc. were categorized as tired before classes in the following results,

- subjects who reported after the test at least two symptoms, such as feeling pressure in the ears or vibrations in the body (i.e., gave 2 or more YES answers to questions 1-6 of the final questionnaire) were recognized as perceiving some changes in exposure conditions,

- subjects who reported at least 2 ailments subjectively related to exposure conditions (i.e., gave 2 or more YES replies to questions 7, 7a-7f on well-being in the final questionnaire) were classified as sensing the impact of exposure conditions.

The analysis, using a gamma coefficient, revealed statistically significant relationships between gender and the majority of replies to the post-exposure questionnaire (Table S1), while only a quite strong negative association was noted between gender and the answer to question 6 of the pre-exposure questionnaire (a gamma coefficient=–0.479, $p < 0.05$), indicating that men more likely slept well before the experiment. The highest positive associations were noted for questions 7a and 7e, which indicates that women suffered more from headaches and tiredness (Table S1).

Table S1. Relationships between gender and outcomes of the post-exposure questionnaire assessed using a gamma coefficient. Data concern all study subjects.

	Answers to the questionnaire	γ coefficient
1	Felt an additional signal, stimulus or other unusual sensation during the classes.	0.024
2	Heard noise, a hum or sound other than the typical background acoustics	0.247*
3	Felt pressure changes in the ears	–0.384*
4	Felt pressure changes in the head	–0.134
5	Felt vibrations in the room	–0.476*
6	Felt vibrations in the body	–0.021
7	Experienced physical or mental discomfort	–0.041
7a	Headache	0.557*
7b	Concentration problem	0.326*
7c	Dizziness	–0.560*
7d	Sleepiness	0.399*
7e	Tiredness	0.617*
	Perceived some changes in exposure conditions	–0.103
	Sensed the impact of some exposure conditions	0.357*
	The total number of feelings subjectively related to exposure conditions	–0.022

The total number of ailments subjectively related to exposure conditions	0.330*

*Significant values of the γ coefficients ($p < 0.05$).

To study the impact of some variables on the perceived changes in exposure conditions and sensed impact on well-being due to exposure conditions, a binary logistic multiple regression was applied with the logistic model expressed as follows:

$$\ln[p/(1-p)]=b_0+b_1x_1+b_2x_2+ \dots +b_nx_n, \quad \text{(S1)}$$

where: p – probability of the outcome, i.e., any perceived changes in exposure conditions or sensed impact of exposure conditions on well-being; $x_1, x_2, \dots, x_n$ are the independent variables included in the model, i.e., gender (males=0, females=1), type of noise conditions (no stimulus=0, reference signal=1, stimulus=2), fatigue before classes (yes/no); $b_0, b_1, \dots, b_n$ – regression coefficients (i.e., the logarithmic values of the odds ratio (OR)). An (OR) with 95% confidence intervals (CI) > 1 indicates a positive correlation between the dependent variable and explanatory variable, while a value < 1 indicates a negative correlation between the dependent variable and explanatory variable. The binary logistic regression revealed that the perceived changes in exposure conditions was positively associated with the type of noise conditions (OR=1.762, 95% CI: 1.141-2.719, p=0.011), while the sensed impact on well-being related to exposure conditions was positively associated with gender (OR=3.445, 95% CI: 1.318-9.001, p=0.012) and fatigue before classes (OR=3.882, 95% CI: 1.789-8.424, p=0.001) (Table S3). The Exp(b1) was 1.762, it means that the reference signal vs background noise conditions as well as the stimulus vs reference signal conditions were associated with a 76% increased risk (OR=1.762) of the perception of changes in exposure conditions (i.e., feeling 2 or more symptoms such as additional sounds, pressure in ears or/and head, vibration in room or/ and in the body, etc.). On the other hand, the odds ratio for the sensation of impact of exposure conditions would increase 3.445 times in the case of women (compared to men) and 3.882 times in the case of people being vs not being tired before classes.

Table S2. Association between PC (any perceived changes in exposure conditions) or SI (the sensed impact of exposure conditions on well-being) (dependent binary variable) and gender, fatigue and type of noise conditions (independent variables) tested using logistic regression.

**Variable**	**Perceived changes in exposure conditions (PC)**		**Sensed impact of exposure conditions on well-being (SI)**	
	**OR (95% CI)**	**p**	**OR (95% CI)**	**p**
Gender	0.954 (0.383-2.375)	0.919	3.445* (1.318-9.001)	0.011
Fatigue before classes	1.285 (0.608-2.718)	0.507	3.882* (1.789-8.424)	0.001
Type of noise exposure	1.762* (1.141-2.719)	0.010	1.127 (0.735-1.726)	0.580
	Chi^2=7.851139 df=3, p=0.0492091, R^2=8.06%, CCR=66.67%		Chi^2=19.86572 df=3, p=0.0001815, R^2=19.09%, CCR=66.67%	

OR – odds ratio. CI – confidence interval.
*Significant associations ($p < 0.05$)

Further statistical analysis, using the gamma coefficient shown in Table S3, revealed some associations between outcomes of the questionnaires and noise parameters. In particular, the following has been found:

- a relatively strong correlation between the total number of various feelings related to exposure conditions and the differences between equivalent C- (or Z-) and A-weighted sound pressure levels (SPLs), and the equivalent-continuous G-weighted SPLs,
- a relatively strong negative correlation between the total number of various feelings related to exposure conditions and the L_{Aeq} level, i.e., the higher the SPLs within the audible frequency range, the lower the perception of any changes in exposure conditions, including infrasound and LFN,
- a significant correlation between the total number of various ailments reported after exposure and the L_{Geq}, L_{Ceq} and $L_{pA,LF}$ levels.

Table S3. A gamma coefficient between the noise parameters and the outcomes of the post-exposure questionnaire.

Noise parameter	Total number of	
	feelings	ailments
	γ coefficient	
Difference between C- and A-weighted equivalent-continuous SPLs, C-A	0.498*	0.122
Difference between Z- and A-weighted equivalent-continuous SPLs, Z-C	0.466*	0.075
G-weighted equivalent-continuous SPL, L_{Geq}	0.312*	0.146*
Low frequency A-weighted SPL, $L_{p A, LF}$**	0.221*	0.177*
A-weighted equivalent-continuous SPL, L_{Aeq}	–0.380*	–0.020
C-weighted equivalent-continuous SPL, L_{Ceq}	0.200*	0.142*

* Significant values of the γ coefficients ($p < 0.05$).

** Low frequency A-weighted sound pressure level ($L_{pA, LF}$), which is determined from the results of frequency analysis using formula (S2).

$$L_{\mathrm{pA,LF}} = 10 \times \log \sum_{f=10\mathrm{Hz}}^{160\mathrm{Hz}} 10^{0,1\times(L_{\mathrm{fieq}}+K_{\mathrm{Af}})} \quad \text{(S2)}$$

where L_f is the measured sound pressure level in 1/3-octave frequency bands from 10 to 160 Hz, and K_{Af} is the value of the A-weighted correction from 10 to 160 Hz.

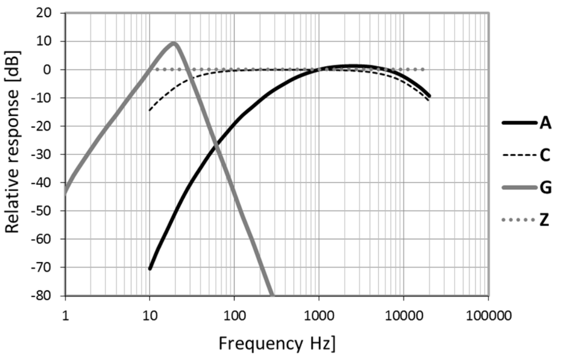

Figure S1. Nominal G-, A-, C- and Z-weighting characteristics according to ISO 7196:1995 and IEC 61672-1:2013.
